# Soluble Immune Checkpoints, Gut Metabolites and Performance Status as Parameters of Response to Nivolumab Treatment in NSCLC Patients

**DOI:** 10.3390/jpm10040208

**Published:** 2020-11-04

**Authors:** Ilaria Grazia Zizzari, Alessandra Di Filippo, Fabio Scirocchi, Francesca Romana Di Pietro, Hassan Rahimi, Alessio Ugolini, Simone Scagnoli, Pamela Vernocchi, Federica Del Chierico, Lorenza Putignani, Aurelia Rughetti, Paolo Marchetti, Marianna Nuti, Andrea Botticelli, Chiara Napoletano

**Affiliations:** 1 Department of Experimental Medicine, Sapienza University of Rome, 00161 Rome, Italy; ilaria.zizzari@uniroma1.it (I.G.Z.); alessandra.difilippo@uniroma1.it (A.D.F.); fabio.scirocchi@uniroma1.it (F.S.); hassan.rahimi@uniroma1.it (H.R.); alessiougolini217@gmail.com (A.U.); aurelia.rughetti@uniroma1.it (A.R.); marianna.nuti@uniroma1.it (M.N.); 2 Department of Clinical and Molecular Medicine, Sant’Andrea Hospital, “Sapienza” University of Rome, 00189 Rome, Italy; francy27@live.it (F.R.D.P.); paolo.marchetti@uniroma1.it (P.M.); 3 Department of Radiological, Oncological and Pathological Science, Policlinico Umberto I, Sapienza University of Rome, 00161 Rome, Italy; simone.scagnoli@uniroma1.it (S.S.); andrea.botticelli@uniroma1.it (A.B.); 4 Area of Genetics and Rare Disease, Unit of Human Microbiome, Bambino Gesù Children’s Hospital, IRCCS, 00165 Rome, Italy; pamela.vernocchi@opbg.net (P.V.); federica.delchierico@opbg.net (F.D.C.); 5 Department of Laboratories, Unit of Parasitology and Area of Genetics and Rare Disease, Unit of Human Microbiome, Bambino Gesù Children’s Hospital, IRCC, 00165 Rome, Italy; lorenza.putignani@opbg.net

**Keywords:** non-small cell lung cancer, soluble immune checkpoints, biomarkers, immune checkpoint inhibitors, gut metabolites, immunotherapy

## Abstract

Patients with non-small cell lung cancer (NSCLC) have been shown to benefit from the introduction of anti-PD1 treatment. However, not all patients experience tumor regression and durable response. The identification of a string of markers that are direct or indirect indicators of the immune system fitness is needed to choose optimal therapeutic schedules in the management of NSCLC patients. We analyzed 34 immuno-related molecules (14 soluble immune checkpoints, 17 cytokines/chemokines, 3 adhesion molecules) released in the serum of 22 NSCLC patients under Nivolumab treatment and the gut metabolomic profile at baseline. These parameters were correlated with performance status (PS) and/or response to treatment. Nivolumab affected the release of soluble immune checkpoints (sICs). Patients with a better clinical outcome and with an optimal PS (PS = 0) showed a decreased level of PD1 and maintained low levels of several sICs at first clinical evaluation. Low levels of PDL1, PDL2, Tim3, CD137 and BTLA4 were also correlated with a long response to treatment. Moreover, responding patients showed a high proportion of eubiosis-associated gut metabolites. In this exploratory study, we propose a combination of immunological and clinical parameters (sICs, PS and gut metabolites) for the identification of patients more suitable for Nivolumab treatment. This string of parameters validated in a network analysis on a larger cohort of patients could help oncologists to improve their decision-making in an NSCLC setting.

## 1. Introduction

The immune checkpoint inhibitors (ICIs) targeting the PD1/PDL1 axis have revolutionized the treatment of cancer. The clinical benefit of these therapies administrated alone or in combination is already well established in several cancer types (melanoma, lung cancer, renal cell carcinoma) [1,2,3]. In other tumors with limited therapeutic options such as glioblastoma, research efforts are focused on possible combination therapies on the tumor microenvironment [4]. These treatments may confer different benefits if combined with standard therapies [5].

In non-small cell lung cancer (NSCLC), the introduction of ICIs has demonstrated a long-term tumor control and improvement of patients’ survival especially in the advanced stage, changing the treatment and the outcome of this severe and often fatal cancer [2].

Despite encouraging results, only 20–30% of NSCLC patients fully respond to these therapies, and predictive biomarkers to enable patient selection have remained elusive [6]. To date, PDL1 expression on tumor tissue (tPDL1) is the only parameter used by clinicians to orient the therapeutic choice. However, the use of tPDL1 as a predictive factor remains challenging: patients with low/negative tPDL1 frequently benefit from anti-PD1 therapy. This is probably due to the dynamic expression of PDL1 especially after chemotherapy [7].

In recent years, several biomarkers associated with tumor biology and patients’ immune fitness have been considered, but several limitations linked to the sampling choice or dynamic expression have hampered the identification of specific parameters usable in the clinical setting [8].

Tumor Mutational Burden (TMB) has been proposed as an independent biomarker of outcome to immunotherapy in NSCLC as well as in other tumors [9]. Checkmate 026 study comparing nivolumab and platinum-based chemotherapy in metastatic NSCLC showed an improvement in progression-free survival (PFS) and objective response rate (ORR) in those patients with high TMB who received immunotherapy [10]. More recently, FDA approved the administration of Pembrolizumab in non-colorectal cancer patients with a high TMB status who experienced failure with prior therapy [11].

Besides tumor biological parameters, it appears that factors that exert a direct or indirect effect on the immune system have to be considered as biomarkers. Recently, the gut microbiota and its metabolites have shown to crucially modulate the anti-tumor immune response and possibly impact the response to ICI therapy [12,13].

Furthermore, the soluble forms of immune checkpoints (sICs) that in vivo are shed or released associated with microvesicles appear to play a crucial role in the modulation of immune fitness of cancer patients [14]. These molecules, such as PDL1, PD1 and CTLA4, affect the efficiency of the immune system contributing to influencing the efficacy of immunotherapy. Their plasma levels are associated with prognosis, response to treatment and ORR [15]. In advanced NSCLC, the serum level of PDL1 alone or in combination with PD1 appeared to be a predictive factor and could identify patients who will benefit from immunotherapy [16,17,18,19]. Although the soluble forms of PDL1 and PD1 are extensively investigated in NSCLC, the role of other sICs as biomarkers was poorly explored.

It is also becoming clear that the “fitness” of the patients is a crucial parameter to be considered for ICI therapy. Indeed, performance status (PS) is a validated clinical parameter for cancer patients. It has been proposed as an independent prognostic factor in advanced NSCLC and as predictor of adverse events and response [20,21]. The link between PS and the immunological fitness was suggested by several data that demonstrated a correlation between PS and the imbalance of circulating T cells in cancer patients [22].

The identification of predictive biomarkers of response represents a key point in the management of NSCLC patients. The interpolation of new diagnostic tools combined with clinical parameters needs to be improved, to identify those patients who could benefit from immunotherapy and to define timing schedules, doses and a new possible combination to decide the proper strategy for each patient.

This is an exploratory study in which we analyzed several soluble immune-related molecules during Nivolumab treatment and the gut metabolomic profile at baseline in 22 NSCLC patients. These factors were combined with clinical parameters such as PS and response to treatment. We showed that the combination of these immunological and clinical factors could be a relevant parameter for the identification of patients who can benefit from ICI therapy. These results can contribute to better designing the roadmap of the immune intervention of NSCLC patients, although further large-scale studies are needed to confirm the usefulness of our findings.

## 2. Results

### 2.1. Patient Characteristics

Twenty-two patients affected by metastatic NSCLC were enrolled in the study. Clinical features are summarized in Table 1. All patients were treated with anti-PD-1 agent nivolumab as second-line (20 patients) or third-line (2 patients) treatment. Median PFS and OS were 5 and 10 months, respectively. Disease progression within 6 months was experienced in half of the patients.

### 2.2. Soluble ICs Are Modulated during ICI Treatments

Several data demonstrated that serum levels of soluble immune mediators were modulated during ICI treatment in cancer patients [15]. To evaluate the impact of Nivolumab treatment on the immune system of NSCLC patients, a panel of 34 immune soluble molecules (sICI, cytokines, chemokines and adhesion molecules) was analyzed in the sera of 22 NSCLC patients collected before nivolumab treatment (T0) and at first clinical evaluation (>T0).

**Figure 1:**
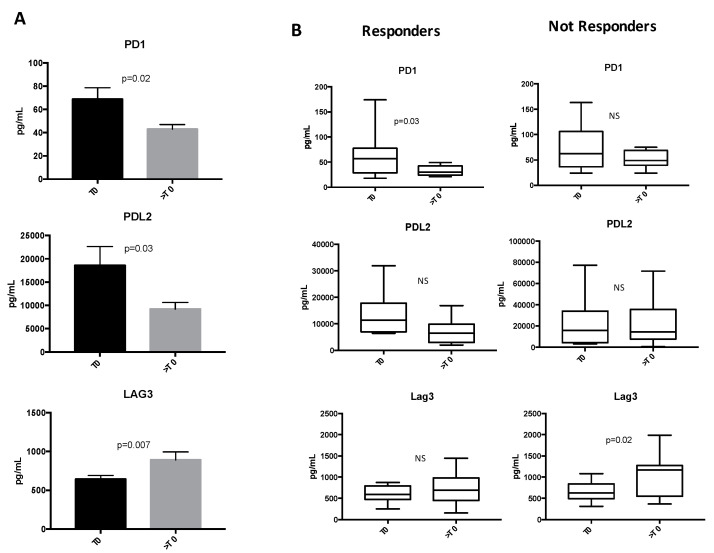
Changes of soluble immune checkpoints (sICs) in non-small cell lung cancer (NSCLC) patients during Nivolumab treatment. (**A**) Analysis of soluble immune check point-related proteins levels evaluated in the entire population of NSCLC patients before the beginning of Nivolumab treatment (T0) and at first clinical evaluation (>T0). The proteins were analyzed by Luminex multiplex assay, and the results are reported as concentration (pg/mL) of sICs present in the patients’ sera. Histograms represent the concentration mean values ±SEM of soluble PD1 (T0 67 ± 10 pg/mL vs. >T0 41 ± 4 pg/mL), PDL2 (T0 18.6 ± 4 ng/mL vs. >T0 8 ± 1.4 ng/mL) and LAG3 (T0 627 ± 50 pg/mL vs. >T0 859 ± 109 pg/mL) at baseline (T0, black histograms) and at first clinical evaluation (>T0, gray histograms). (**B**) Box plots of PD1, PDL2 and LAG3 in responding (R) and non-responding (NR) patients between nivolumab initiation (T0) and the first clinical evaluation (>T0). The lines in the box show the median values. The error bars represent the minimum and the maximum values of sICs concentration (pg/mL). A Student’s paired *t*-test was used to compare the differences between T0 and >T0. *p* values ≤ 0.05 were considered significant. NS = not significant.

Among the immune molecules analyzed, PD1, PDL2 and the co-inhibitory LAG3 receptor were dynamically modulated during Nivolumab treatment (Figure 1), while no significant changes were observed for the other tested immune-molecules (Table S1). PD1 and its ligand PDL2 significantly decreased during Nivolumab treatment, while LAG3 levels increased (Figure 1A). When these profiles were analyzed in regard to clinical response, it appeared that PD1 decrease was observed in the R patient cohort and not in NR group (Figure 1B) suggesting that this modulation is not ascribable to the binding between soluble PD1 and Nivolumab. Conversely, LAG3 levels significantly increased in NR patients. The decrease in PDL2 observed during ICI therapy seemed not to be associated with the response.

### 2.3. Low Levels of sICs Are Associated with Clinical Response in NSCLC Patients

**Figure 2:**
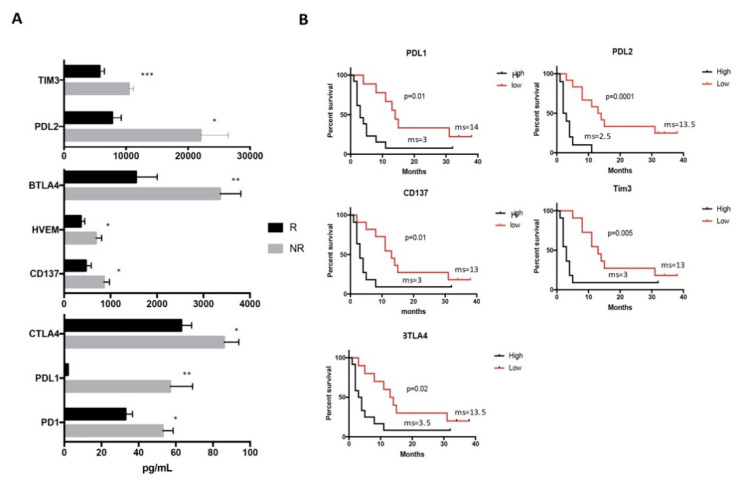
Levels of sICs evaluated in R and NR patients at first clinical evaluation (>T0) and their correlation with duration of response. (**A**) Soluble immune checkpoint-related molecules were evaluated in the serum of responding (R, black histograms) and non-responding (NR, black histograms) patients after three months of the beginning of Nivolumab treatment by Luminex multiplex assay. Histograms represent the concentration mean values ± SEM of PD1 (R 31 ± 4 pg/mL vs. NR 53 ± 6 pg/mL), PDL1 (R 1.7 ± 0.06 pg/mL vs. NR 57 ± 12 pg/mL), CTLA4 (R 61 ± 5 pg/mL vs. NR 86 ± 8 pg/mL), CD137 (R 429 ± 113 pg/mL vs. NR 859 ± 117 pg/mL), HVEM (R 332 ± 82 pg/mL vs. NR 687 ± 124 pg/mL), BTLA4 (R 1.4 ± 0.4 ng/mL vs. NR 3.3± 0.4 ng/mL), PDL2 (R 7.3 ± 1.4 ng/mL vs. NR 22 ± 4.4 ng/mL) and Tim3 (R 5.4 ± 0.8 ng/mL vs. NR 10 ± 0.7 ng/mL) at >T0. A Student’s unpaired *t*-test was used to compare the difference between R and NR patients. *p* values ≤ 0.05 were considered significant. * *p* < 0.05, ** *p* < 0.01, *** *p* < 0.001. (**B**) Survival analysis carried out at >T0 in NSCLC patients with high and low levels of sICs. The concentration median values of PDL1, PDL2, CD137, Tim3 and BTLA4 were used to define patients with high and low levels of sICs. The concentration median values are the following: PDL1 (20 pg/mL), PDL2 (7.7 ng/mL), CD137 (624 pg/mL), Tim3 (8.1 ng/mL) and BTLA4 (2.2 ng/mL). Long-rank tests were used to compare the duration of response between the two groups. ms = median survival.

It is interesting to note that at the beginning of therapy, similar levels of sICs were detected in both R and NR patient groups (data not shown), while at first clinical evaluation, the R patients displayed lower levels of sICs as compared to NR (Figure 2A).

We found that the concentrations of soluble PD1 as well as both its ligands PDL1 and PDL2 were significantly lower in R than NR patients. Additionally, the inhibitory BTLA4 and its receptor HVEM displayed lower levels in R patient group. A similar pattern was also found for the inhibitory molecules Tim3 and CTLA4. All these molecules contributed to negatively modulating the activation of T cell response both as soluble forms and as membrane-bound molecules [23,24]. Interestingly, the costimulatory molecule CD137 is also low in R patients. This protein, which acts as a T cell activator and membrane-bound molecule, in soluble form, prevents the activation of T cells and the maturation of antigen-presenting cells such as dendritic cells [25].

These results strongly suggest that the response to Nivolumab treatment is strongly associated with low levels of sICs as measured 3 months from the start of the therapy.

To better understand the significance of such experimental evidence with the clinical outcome of the patients, we verified the association between these sICs and the duration of clinical response. For this purpose, the concentration median values of sICs were calculated and patients were stratified accordingly. Results confirmed that patients with low levels of PDL1, PDL2, CD137, Tim3 or BTLA showed a long clinical response (Figure 2B), suggesting the possible role of these molecules as biomarkers of response to treatment.

### 2.4. sICs Are Differently Modulated According to ECOG PS Scale

**Figure 3:**
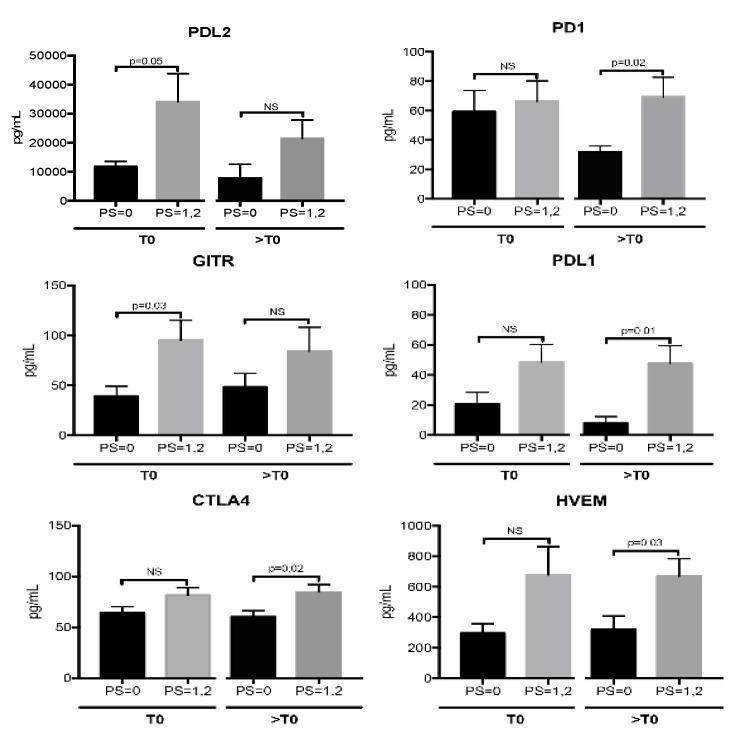
Levels of soluble immune check point-related molecules evaluated in NSCLC patients scored as performance status (PS) = 0 and PS = 1,2 before the beginning of nivolumab treatment (T0) and at first clinical evaluation (>T0). Histograms represent the concentration mean values of sICs ± SEM detected in the serum of patients with PS = 0 (9 patients) (black histograms) and PS = 1,2 (13 patients) (grey histograms) by Luminex multiple assay. The median values of sICs ± SEM at T0 are as follows: PDL2: PS = 0 vs. PS1,2, 12 ± 2 vs. 34 ± 10 ng/mL; GITR: PS = 0 vs. PS1,2, 39 ± 10 vs. 95 ± 20 pg/mL; CTLA4: PS = 0 vs. PS1,2, 64 ± 6 vs. 81 ± 7 pg/mL; PD1 PS = 0 vs. PS1,2, 59 ± 14 vs. 71 ± 14 pg/mL; PDL1: PS = 0 vs. PS1,2, 21 ± 7,5 vs. 49 ± 12 pg/mL; HVEM: PS = 0 vs. PS1,2, 296 ± 62 vs. 677 ± 185 pg/mL. At >T0, the mean values of sICs ± SEM are: PDL2: PS = 0 vs. PS1,2, 8 ± 1,5 vs. 21 ± 7 ng/mL; GITR: PS = 0 vs. PS1,2, 48 ± 13 vs. 84 ± 23 pg/mL; CTLA4: PS = 0 vs. PS1,2, 60 ± 6 vs. 84 ± 7.3 pg/mL; PD1 PS = 0 vs. PS1,2, 32 ± 4 vs. 69 ± 13 pg/mL; PDL1: PS = 0 vs. PS1,2, 8 ± 4 vs. 48 ± 12 pg/mL; HVEM: PS = 0 vs. PS1,2, 319 ± 90 vs. 668 ± 115 pg/mL. A Student’s unpaired *t*-test was used to compare the differences of sIC levels between patients with PS = 0 and PS = 1,2 evaluated at T0 and at >T0. *p* values ≤ 0.05 were considered significant.

Soluble ICs were further analyzed stratifying the patients according to the Eastern Cooperative Oncology Group (ECOG) PS scale into 2 groups: PS = 0 and PS = 1,2. Among the patients enrolled in this study, 9 were classified as PS = 0 and 13 as PS = 1,2 (11 patients with PS = 1; 2 patients with PS = 2). Results showed that patients scored as PS = 0 had decreased levels of PD1 following nivolumab treatment (PD1: T0 59 ± 14 pg/mL vs. >T0 32 ± 4 pg/mL, *p* = 0.04). These levels remained similar in PS = 1,2 patients (PD1: T0 71 ± 14 pg/mL vs. >T0 69 ± 13 pg/mL, *p* = 0.8). Moreover, the analysis displayed a trend of association between low sIC levels and PS = 0 (Figure 3). This group of patients showed a better immune fitness before the beginning of therapy compared to the PS = 1,2 group showing low levels of the inhibitory molecules PDL2 and GITR. At first clinical evaluation, PD1, PDL1, CTLA4 and HVEM were less abundant in PS = 0 patients compared to PS = 1,2 group.

These data suggest that Nivolumab treatment appears to be more efficient in patients with PS = 0, decreasing PD1 and maintaining low levels of immunosuppression.

### 2.5. Responding Patients Have a High Proportion of Eubiosis-Associated Gut Metabolites

Several data demonstrated that specific gut microbiota and metabolome profiles have been associated with eubiosis or dysbiosis status [26]. These two conditions severely impact the immunological fitness of cancer patients influencing the response to treatment [12].

To analyze the gut microbiota and the proportion of eubiosis/dysbiosis-associated gut metabolites in R and NR patients, stool samples derived from 11 out of 22 NSCLC patients (6 R and 5 NR) were collected at T0 and analyzed. The metagenomics data revealed that no cluster formation was found in R and NR patients, suggesting that no specific cluster was associated with clinical response (see Table S2).

**Figure 4:**
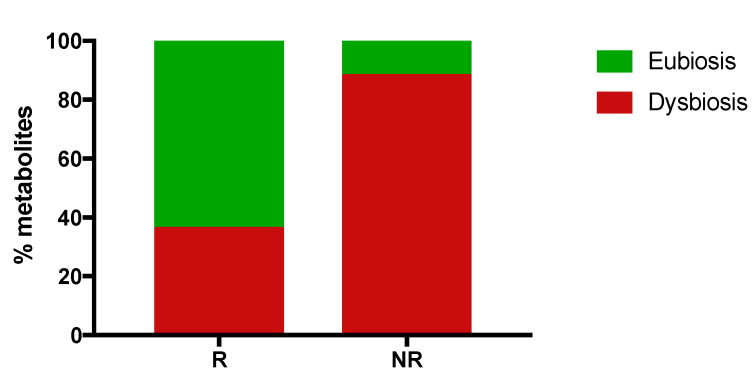
Percentage of eubiosis/dysbiosis gut metabolites in responding (R) and non-responding (NR) patients evaluated before the beginning of nivolumab treatment (T0). Gut metabolites were evaluated in the fecal samples of 11 NSCLC patients. Volatile organic compounds were analyzed by gas chromatography-mass spectrometry, while non-volatile metabolites were analyzed by proton nuclear magnetic resonance spectroscopy. In R patients, 14 metabolites were found, 9 related to eubiosis and 5 to dysbiosis. Non-responding patients (NR) showed 17 metabolites, 2 related to eubiosis and 15 to disbiosys. The histograms represent the percentage of eubiosis- (in green) and dysbiosis-(in red) associated gut metabolites evaluated, considering the 14 and 17 metabolites found in R and NR patients as 100%, respectively.

Furthermore, the analysis of the gut metabolome profile showed a set of 114 metabolites, 67 (59%) volatile organic compounds (VOCs) and 47 (41%) non-volatile (Table S3). Among these compounds, 42 were related to eubiosis or dysbiosis (14 and 28, respectively). In particular, VOCs showed the eubiosis related metabolites belonging to a chemical class of short chain fatty acids (SCFAs) (i.e., butyric, proprionic, acetic and pentanoic) and terpenes; on the contrary, the metabolites probably associated with dysbiosis were aldehydes (i.e., butanal 3-Methyl, benzeneacetaldehyde), alcohols (i.e., ethanol, 2-Octanol) and phenols. Evaluating the concentration average values of these 42 metabolites in R and NR patients, 31 of them showed a difference of at least two-fold in their concentration between the two groups and were further analyzed (Table S4 and Figure 4). In R patients, a total of 14 compounds were found, 9 (64%) potentially related to eubiosis and 5 (36%) to dysbiosis, while NR patients showed 17 metabolites, 2 (12%) might be associated with eubiosis and 15 (88%) with dysbiosis.

## 3. Discussion

Advanced NSCLC is among the tumors that have most benefited from the introduction of anti-PD1 treatment as second-line therapy [27]. Moreover, combination therapy with Nivolumab plus Ipilimumab (anti-CTLA4) demonstrated an increase in PFS in those patients with high TMB, adding a further therapeutic strategy in this setting of patients [9].

The target of these drugs is not just the tumor cells, but the patient’s immune system. All cancer patients are potentially treatable with ICI, although a high percentage of them does not benefit from these types of treatments. Among the major challenges is now to understand the rationale for using ICIs, which appears to have several differences in terms of clinical efficacy. For this reason, the identification of predictive markers of response is a challenging approach for drug selection to obtain the best clinical benefit.

Several studies demonstrate that the identification of a single biomarker fails to discriminate “fit” from “unfit” patients due to the complexity of the interaction between tumor and the immune system. This interaction is also strongly influenced by several other mechanisms that come into play when immunotherapy is administered [28]. Checkpoint inhibitors directly contribute to modifying the activation state of the immune cells, thus resulting in the modulation of other immune-related parameters that contribute to influencing the immune landscape of cancer patients. Among the possible mechanisms influenced by ICIs treatment, sICs represent a key point in regulating the patient’s immune system. While the function of ICs as membrane-bound molecules is well defined in the regulation of immune response [29], their activity as soluble forms remains uncertain and probably strictly correlated with the immunological milieu. To date, soluble PDL1 and PD1 remain the two most studied molecules in NSCLC. Soluble PDL1 has been clearly defined as a negative regulator [14]. In lung cancer, high levels of PDL1 are associated with poor prognosis, shorter OS and the presence of abdominal metastasis [16,30]. More recently, soluble PDL1 serum concentration has been associated with a high metabolic tumor burden, suggesting that the levels of PDL1 may reflect the expansion of tumor volume and tumor lysis during ICI treatment [31]. More discussed is the role of PD1 as a soluble form. Several studies ascribed a significant improvement in the antitumor immunity to PD1, hypothesizing that the binding between soluble PD1 and the membrane-bound PDL1/PDL2 might prevent T cell inhibition [32]. Moreover, increased or stable levels of soluble PD1 correlate with longer PFS and OS in NSCLC Nivolumab-treated patients after two cycles of therapy [19]. On the contrary, other studies describe soluble PD1 as a negative regulator: low levels of PD1 favor the activation of the immune system inducing the maturation of dendritic cells and decreasing the threshold of T cell activation [33].

Among the immune modulators analyzed in this study, only sICs appear to be related to Nivolumab treatment and clinical parameters such as response to therapy and PS. We observed that Nivolumab modulates the release of sICs maintaining low levels of immunosuppression in those patients with presumably a fully active immune system (responding patients). Patients with better clinical outcomes showed decreasing levels of PD1 between the beginning of therapy and the first clinical evaluation. This significant decrease observed only in the responding group could be a consequence of the saturation of this molecule, which might be incomplete in non-responding patients due to upregulation as escape mechanisms to anti-PD1 treatment. However, our results cannot exclude that a minor reduction in PD1 levels could be present also in non-responding patients if a greater sample size had been analyzed. This decrease was not observed for the other sICs analyzed in this study, including PDL1, even if their levels were maintained lower in the responding patients. Moreover, the main difference in soluble IC levels found in this study was associated with the response and not the basal values. These preliminary results highlight the importance of monitoring these soluble molecules to follow the response to immunotherapy and to improve patient selection and represent the basis for further studies to explore the biological mechanism of ICI treatment.

The efficiency of the immune system strongly impacts the clinical condition of cancer patients. Performance status represents a clinical measure to establish cancer progression and how this progression affects the living activity of oncology patients. Most studies do not enroll patients with poor PS (PS ≥ 2) because this is a negative prognostic factor for response to treatment and survival and a predictive factor of adverse events [20]. This group of patients is heterogeneous and with moderate or severe comorbidities, frequently requiring the use of antibiotics or corticosteroid which influence the activation of the immune response and the response to ICI [34,35]. Solid studies regarding the benefit of ICIs in cancer patients with poor PS remain uncertain. Data from a recent meta-analysis reports that ICIs improve survival irrespective of patients’ PS [36,37]. A small phase II trial conducted on NSCLC patients under pembrolizumab treatment demonstrated that patients with PS = 0,1 and PS = 2 have similar efficacy and grade of toxicity from ICI therapy [38]. In our study, sICs seem to be modulated by nivolumab treatment according to patients’ PS. Patients with a PS = 0 were linked to a better immunological fitness at the beginning of therapy. Nivolumab seems to perform its immunoregulatory function more effectively in this setting of patients reducing PD1 and maintaining several sICs at low levels with immunosuppressive functions. Similar results were also obtained when the two patients with PS = 2 were excluded by the analysis (data not shown), highlighting a difference in the immunosuppression status between patients with PS = 0 and PS = 1. This is a crucial issue: indeed, these two groups of patients are enrolled in ICI clinical trials without distinctions; the lack of effectiveness of ICIs in a high percentage of patients could also be ascribed to different levels of immunosuppression among patients. The association between PS = 0 and low sIC could be obvious, as it is well known that these patients respond better to ICI. However, this is the first work that demonstrates a correlation between sIC levels and patients’ PS, which, although preliminary, may serve as a starting point for future works aimed at deepening the association between these two parameters.

In addition, an important district that could influence the immune systems response is represented by gut microbiota and its metabolomic profile. The functionality of gut microbiota also affects the immune fitness and the relationship with the response to immunotherapy and the delicate balance between the tumor and immune system.

The data obtained revealed that R patients have a more wellness-oriented gut metabolomic profile which could be used as an important tool to identify patients with a better clinical outcome.

Specific gut metabolomic pathways were found and were clearly associated with early progression and long response to therapy in the patients enrolled in this study [13]. Here, we investigated the metabolomic profile of R and NR patients recognizing a role probably associated with dysbiosis or eubiosis.

Responder patients showed higher levels of SCFA which were produced from the indigestible carbohydrates as fibers by microbes and appear to be a key intermediary of the beneficial effects caused by the gut microbiome. SCFA production is essential for integrity by the regulation of the luminal pH, action on mucosal immune function and mucus production, and they also provide energy for epithelial cells. In addition, the host metabolic health is directly modulated from the SCFA through a range of mechanisms tissue-specific related to immunomodulation, appetite regulation, energy expenditure and glucose homeostasis [39]. Terpenes among other phytochemicals such as polyphenols, carotenoids, phytosterols/phytostanols possess anti-inflammatory and antioxidant properties that could positively influence the GM [40].

NR patients show a prevalence of dysbiosis-associated metabolites compared to responders, suggesting an imbalanced microbiota metabolism at baseline.

Alcohols seems to be a mediator to the development of non-alcoholic steatohepatitis (NASH) [41,42], and it was hypothesized that when gut microbes produce alcohols they may cause endotoxemia [43]. Therefore, the high levels of alcohols (i.e., ethanol, 2-Octanol) characterized an “unbalanced” microbial ecology gut level.

Aldehydes could promote mutagenesis and could be associated with bowel cancer, but the toxic effects of higher aldehydes have received much less attention [44].

Experimental studies have shown an ability to damage cellular structure and increase permeability. It is likely that these changes could increase the susceptibility of the colonic epithelium to luminal carcinogens [45]. Due to the strict correlation between bacterial metabolism and immune fitness, it is conceivable to believe that the presence of eubiosis-related compounds in responding patients contributes to maintaining optimal the wellness of the immune fitness, thus resulting in a better and durable response to nivolumab treatment.

**Figure 5:**
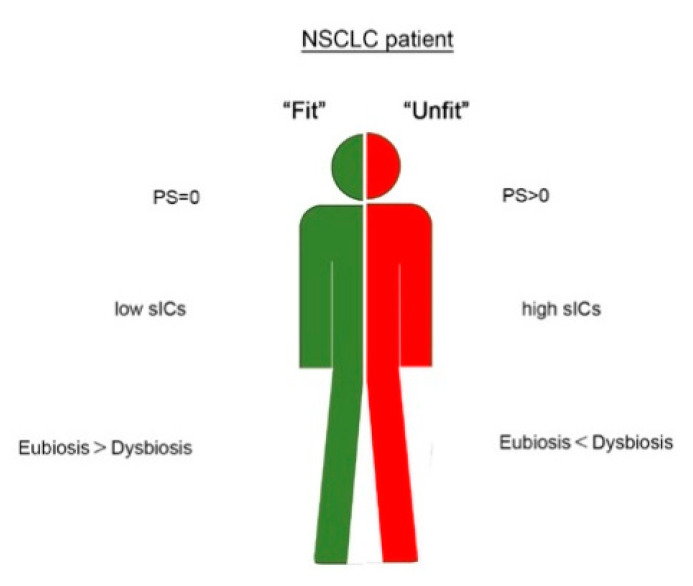
Schematic representation of a potential string of predictors useful to discriminate fit (in green) and unfit (in red) NSCLC patient.

A limitation of the study is the sample size. A greater population could have highlighted smaller differences in the concentration of other sICs. However, the preliminary results showed in this study highlight the possible role of several circulating molecules (PDL1, PDL2, CD137, Tim3 and BTLA4) as parameters of response in NSCLC patients. These molecules combined with patients’ PS and the composition of gut metabolites may have the potentiality to identify the immunological and clinical profile of NSCLC patients more suitable for immunotherapy treatment (Figure 5). Due to the urgent need to increase the number of responding patients treated with immunotherapy, we believe that these results could be helpful for clinicians to improve their decision-making in NSCLC patients and could be the basis of future research in other cancers treated with ICI. The aim is now to translate these findings into large-scale studies of patients using a network-based approach that includes clinical and experimental data.

## 4. Material and Methods

### 4.1. Patients

In this study, patients with stage IV NSCLC treated with nivolumab at Sant’Andrea Hospital were enrolled. Criteria of inclusion were: age > 18 years; Eastern Cooperative Oncology Group (ECOG) performance status ≤ 2; histologically-documented diagnosis of NSCLC; adequate pulmonary, cardiac, liver, renal and bone marrow function; measurable disease; patients with symptomatic and stable central nervous system metastases were eligible. Criteria of exclusion were: symptomatic interstitial lung disease and any other significant comorbidity; autoimmune disease; prior treatment with immune-stimulatory antitumor agents, including checkpoint-targeted agents; systemic immunosuppression. Written informed consent was given by all patients. The study was conducted in accordance with good clinical practice guidelines and the declaration of Helsinki. The Institutional Ethics Committee of the two institutions involved agreed to the final version of the protocol (Ethical Committee: Policlinico Umberto I and Azienda Ospedaliera S. Andrea, “Sapienza” University of Rome, RIF.CE: 4181).

### 4.2. Treatment, Efficacy and Safety Assessments

A standard dose of 3 mg/kg every 2 weeks of Nivolumab was administered intravenously until disease progression or development of unacceptable toxicity. Tumor response was evaluated at week 12 and every 12 weeks thereafter until disease progression using immune-related Response Evaluation Criteria in Solid Tumors Criteria (i-RECIST) and classified according to disease control as complete response, partial response, stable disease and progressive disease. The radiological assessment was evaluated with CT scan and PET or MR if indicated. Safety assessments were carried out at day 1 of each cycle until the end of therapy, and toxicities were classified according to the National Cancer Institute Common Terminology Criteria for Adverse Events (version 4.0). The time frame between the start of Nivolumab treatment and the first documented tumor progression or death by any cause is called progression-free survival (PFS). Overall survival (OS) defines the amount of the time from the beginning of nivolumab to death by any cause.

Responder patients were defined as those experiencing complete response, partial response and stable disease within 6 months from the beginning of nivolumab treatment.

### 4.3. Serum and Fecal Collection

Blood samples derived from 22 NSCLC patients were collected using BD Vacutainer Plus Plastic Serum tubes (Becton Dickinson, NJ, USA). The tubes were centrifuged for 10 min at 1800 rpm, and patients’ sera were isolated and cryopreserved (−20 °C) until use. Blood withdrawals were carried out before Nivolumab treatment (T0) and after 6 cycles of therapy (>T0) corresponding to the first clinical evaluation (12 weeks). Four non-responding patients experienced disease progression within 3 months. Their blood samples were analyzed after 2 or 3 cycles of treatment.

Fecal samples derived from 11 patients were collected at baseline and stored at −20° before analysis.

### 4.4. Measurement of Soluble Immune Mediators in the Serum

The level of soluble immune mediators in the serum of NSCLC was detected before and during Nivolumab treatment using the Inflammation 20-Plex Human ProcartaPlex Panel and Immuno-oncology Checkpoint 14-Plex Human ProcartaPlex Panel (both from ThermoFisher Scientific, Waltham, MA, USA) according to the manufacturers’ instruction. The concentration of 34 immune mediators was evaluated (BTLA, GITR, HVEM, IDO, LAG-3, PD-1, PD-L1, PD-L2, TIM-3, CD28, CD80, CD137, CD27, CD152, sE-Selectin, GM-CSF, ICAM/CD54, IFNα, IFNγ, IL1α, IL1β, IL4, IL6, IL8, IL10, IL12p70, IL13, IL17A/CTLA8, IP10/CXCL10, MCP1/CCL2, MIP1α/CCL3, MIP1β/CCL4, sP-Selectin, TNFα) by Luminex multiple assay and analyzed using Bioplex Manager MP software (Bio-Rad, Hercules, CA, USA).

### 4.5. Targeted Metagenomic on Fecal Microbiota

DNA was extracted from stool samples by a QIAmp Fast DNA Stool mini kit (Qiagen, Hilden, Germany), following the manufacturer’s instructions. The bacterial DNA library was obtained by the amplification of 16S rRNA variable region V3-V4 (~460 bp) following the MiSeq rRNA Amplicon Sequencing protocol (Illumina, San Diego, CA, USA). The pooled library was sequenced on an Illumina MiSeqTM platform according to the manufacturer’s specifications. Obtained raw reads, after quality and length trimming and chimera checking, were analyzed by Qiime v1.8.(http://qiime.org/1.4.0/) [46]. Operational Taxonomic Units (OTUs, Chicago, IL, USA) with a 97% clustering threshold of pairwise identity and representative sequences were aligned using PyNAST v.0.1. (https://biocore.github.io/pynast/) [47] and matched against Greengenes 13_08 database [48]. An OTU table was filtered, retaining all OTUs that had at least a 0.01% total abundance in the table and removing all OTUs present in less than 25% of samples.

### 4.6. Gut Microbiome Metabolomics Profiling

The gut metabolome profile was characterized for 11 NSCLC patients in order to analyze volatile and non-volatile metabolites. For volatile organic compound (VOC) detection, stool samples were analyzed with gas chromatography-mass spectrometry (GC-MS) to detect volatile organic compounds (VOCs), by using the carboxen-polydimethylsiloxane coated fiber (CAR-PDMS) (85 μm) and the manual solid-phase microextraction (SPME) holder (Supelco Inc., Bellefonte, PA, USA) according to Vernocchi et al. [42]. Run conditions were previously reported by Botticelli et al. [13]. The chromatograms were integrated and identified compared to fragment pattern presents in the mass spectral NIST library (version 2.2, NIST 14MS database; National Institute of Standards and Technology, Rockville, MD, USA), with the literature [44] and also followed by manual visual inspection. Quantitative data compounds were obtained by interpolation of the relative areas vs. internal standard (IS) area expressed as ppm (mg/kg).

Determination of non-volatile metabolites was performed by nuclear magnetic resonance spectroscopy (NMR) analysis; the stools were processed to obtain fecal waters as described by Brasili et al. [49]. Subsequently, to sample collection, 2 out of 11 NSCLC samples were excluded for an inadequate sample amount. The pipelines and the NMR analyses were performed according to Brasili et al. [50] and Botticelli et al. [13]. Moreover, the assignment was confirmed according to the Human Metabolome Data Base [51] and our own laboratory database. 1D 1H NMR spectra were processed and quantified (µmol/g) according to Botticelli et al. [13].

### 4.7. Statistical Analysis

Statistical analysis was performed using Graphpad Prism version 7 (Graphpad Software, Inc., San Diego, CA, USA). Descriptive statistics (average and standard error of the mean (SEM)) were used to describe different groups of continuous data. A Student’s *t*-test was used to compare groups of continuous variables. Groups of categorical data were compared using the Fisher’s exact test. Survival curves were plotted employing the Kaplan–Meier method and compared by using the log-rank test. The significant level was defined as *p*-value ≤ 0.05.

## Figures and Tables

**Table 1:** Clinical feature of NSCLC patients.

Patient Characteristics	N (%)
**Age**	
≤65	12 (55)
>65	10 (45)
**Gender**	
Male	16 (73)
Female	6 (27)
**Smoking**	
Yes	19 (86)
No	3 (14)
**Histology**	
Adenocarcinoma	4 (18)
Sq. Cell carcinoma	18 (82)
**Site of metastasis**	
Lymph nodes	15 (68)
Lung	20 (91)
Liver	3 (14)
Brain	4 (18)
Bone	4 (14)
Other	5 (23)
**N° of affected organs**	
1	5 (23)
2	10 (45)
3	4 (18)
>3	2 (9)
**cT before Nivolumab**	
X	1 (4)
0	4 (18)
1	2(9)
2	0
3	6 (27)
4	9 (41)
**cN before Nivolumab**	
0	9 (41)
1	11 (50)
2	2 (9)
**Treatment lines**	
2	20 (91)
>2	2 (9)
**Response to Nivolumab**	
Yes	11 (50)
No	11 (50)

## Supplementary Materials

The following are available online at https://www.mdpi.com/2075-4426/10/4/208/s1, Table S1: Immune molecules analyzed in the serum of NSCLC patients at T0 and >T0 with no significant difference; Table S2: Gut microbiota analysis; Table S3: Volatile and non-volatile metabolites evaluated in R and NR NSCLC patients; Table S4: volatile and non-volatile dysbiosis- and eubiosis-associated metabolites evaluated in R and NR NSCLC patients.
