# Supplementary material for: Soluble Immune Checkpoints, Gut Metabolites and Performance Status as Parameters of Response to Nivolumab Treatment in NSCLC Patients
Source: J Pers Med. 2020 Nov 4;10(4):208. doi: 10.3390/jpm10040208 (PMC7712566; doi:10.3390/jpm10040208)
Supplement: Supplementary file 1 [file jpm-10-00208-s001.zip › supplementary figure/Suppl Table 4 .pdf]

Table S4: volatile and non-volatile dysbiosis- and eubiosis-associated metabolites evaluated in R and NR NSCLC patients

Volatile metabolites		
COMPOUNDS (mg/kg)	Average value NR	Average value R
1-Butanamine, N-methyl-	1,259	0,000
2-Butanol, 3-methyl-	1,576	0,000
2-Ethyl-1-hexanol	19,866	0,000
2-Methyl-3-pentanol	20,690	0,000
n-Hexylmethylamine	9,183	0,000
1-Hexanol	18,891	8,973
2-Hexanol	72,105	9,133
2-Propanamine	12,672	1,141
Benzaldehyde	316,528	66,537
Benzeneacetaldehyde	37,536	11,120
Ethanol	93,410	1,959
Furfural	32,416	3,217
Methoxyacetic acid, 3-tridecyl ester	17,571	0,242
p-Cresol	2001,941	535,740
Phenol	111,067	49,843
1-Propanamine, N,2-dimethyl-	0,000	0,815
2-Octanol	0,000	0,701
3-Methyl-3-buten-1-ol	0,000	0,656
Butanal, 3-methyl-	0,000	32,247
heptanal	0,000	0,457
Butyric acid	0,000	34,523
Caryophyllene	0,000	6,495
g-Terpinene	0,000	0,272
p-Cymene	0,000	28,302
Pentanoic acid	0,000	25,840
3-Carene	0,505	12,293
Anethole	8,370	0,000
Eicosane	0,792	0,000

Non-volatile metabolites		
**COMPOUNDS (μmol/g)**	Average value NR	Average value R
Valerate	0,817122999	1,638736196
Butyrate	2,780920969	7,060359011
Propionate	2,581718723	9,205996028

Dysbiosis-associated metabolites (red)
Eubiosis-associates metabolites (green)
