# Supplementary material for: Soluble Immune Checkpoints, Gut Metabolites and Performance Status as Parameters of Response to Nivolumab Treatment in NSCLC Patients
Source: J Pers Med. 2020 Nov 4;10(4):208. doi: 10.3390/jpm10040208 (PMC7712566; doi:10.3390/jpm10040208)
Supplement: Supplementary file 1 [file jpm-10-00208-s001.zip › supplementary figure/Table S1.pdf]

Table S1: Immune molecules analyzed in the serum of NSCLC patients at T0 and >T0 with no significant difference

	T0	>T0	p value
	pg/mL	pg/mL	
**IL1a**	383	489	0.3
**IL1b***			NA
**IL4**	1598	2216	0.2
**IL6***			NA
**IL8**	816	929	0.6
**IL10**	416	423	0.8
**IL12**	891	1004	0.5
**IL13***			NA
**IL17***			NA
**IFNg**	1151	1357	0.2
**IFNa**	86	109	0.3
**TNFa**	3133	3337	0.3
**GITR**	45	59	0.6
**CCL2**	3835	4036	0.5
**CCL3**	847	983	0.3
**CCL4**	3649	4188	0.2
**GMCSF***			NA
**ICAM1**	396094	466877	0.4
**IP10**	1635	1881	0.3
**P-selectin**	1100905	1154777	0.4
**E-selectin**	312378	346875	0.1
**CD27***			NA
**CD28**	1549	1133	0.2
**CD80**	272	249	0.4
**CD137**	632	644	0.9
**BTLA4**	2332	2400	0.9
**CTLA4**	72	73	0.9
**IDO***			NA
**HVEM**	367	509	0.2
**PDL1**	32	33	0.9
**Tim3**	7815	8093	0.7

NA: not applicable

*concentration value not measurable
