# Supplementary material for: Soluble Immune Checkpoints, Gut Metabolites and Performance Status as Parameters of Response to Nivolumab Treatment in NSCLC Patients
Source: J Pers Med. 2020 Nov 4;10(4):208. doi: 10.3390/jpm10040208 (PMC7712566; doi:10.3390/jpm10040208)
Supplement: Supplementary file 1 [file jpm-10-00208-s001.zip › supplementary figure/Table S2.pdf]

Table S2: Gut microbiota analysis

Order	Family	OTUs*	NR_average value	R_average value
Bacteroidales	Bacteroidaceae	Bacteroides	0,04567	0,09390
Clostridiales	Lachnospiraceae	Lachnospiraceae	0,02488	0,03161
Clostridiales	Ruminococcaceae	Ruminococcus	0,01234	0,01627
Bacteroidales	Bacteroidaceae	Bacteroides_plebeius	0,00156	0,01578
Bacteroidales	Prevotellaceae	Prevotella_copri	0,00000	0,01528
Bacteroidales	Prevotellaceae	Prevotella	0,00218	0,03149
Coriobacteriales	Coriobacteriaceae	Coriobacteriaceae	0,00369	0,01134
Bacteroidales	Rikenellaceae	Rikenellaceae	0,00278	0,03813
Clostridiales	Lachnospiraceae	Coprococcus	0,00366	0,05241
Clostridiales	Lachnospiraceae	Ruminococcus_gnavus	0,00281	0,01239
Clostridiales	Ruminococcaceae	Ruminococcus_bromii	0,03820	0,03304
Lactobacillales	Lactobacillaceae	Lactobacillus	0,01564	0,01506
Lactobacillales	Streptococcaceae	Streptococcus	0,14144	0,03838
Clostridiales	Ruminococcaceae	Ruminococcaceae	0,15676	0,13845
Clostridiales	Ruminococcaceae	Faecalibacterium_prausnitzii	0,02094	0,01563
Clostridiales	Ruminococcaceae	Oscillospira	0,01575	0,01035
Enterobacteriales	Enterobacteriaceae	Enterobacteriaceae	0,08424	0,07599
Verrucomicrobiales	Verrucomicrobiaceae	Akkermansia_muciniphila	0,14429	0,09120
Methanobacteriales	Methanobacteriaceae	Methanobrevibacter	0,03610	0,00588
Bifidobacteriales	Bifidobacteriaceae	Bifidobacterium_longum	0,01266	0,00601
Bacteroidales	Bacteroidaceae	Bacteroides_fragilis	0,01126	0,00700
Enterobacteriales	Enterobacteriaceae	Citrobacter	0,01081	0,00018
Clostridiales	Lachnospiraceae	Dorea	0,01149	0,00323

* OTUs= operational taxonomic unit
